# Characterization of dilated cardiomyopathy using tissue phase mapping and extracellular volume measurement

**DOI:** 10.1186/1532-429X-15-S1-P105

**Published:** 2013-01-30

**Authors:** Lewis C Sommerville, Jacob Fluckiger, Michael Markl, Jeremy Collins, Shivraman Giri, James Carr, Keyur Parekh, Amita Goyal

**Affiliations:** 1 Radiology, Northwestern Memorial Hospital, Chicago, IL, USA; 2 Siemens Healthcare, Chicago, IL,USA

## Background

Non-ischemic dilated cardiomyopathy (DCM) is a relatively common cause of left ventricular dysfunction. Microscopic scar may be a cause of regional and global left ventricular dysfunction in DCM patients. The aim of this study was to evaluate changes in regional myocardial structure, function, and dyssynchrony using a novel non-invasive MR imaging protocol.

## Methods

**Figure 1:**
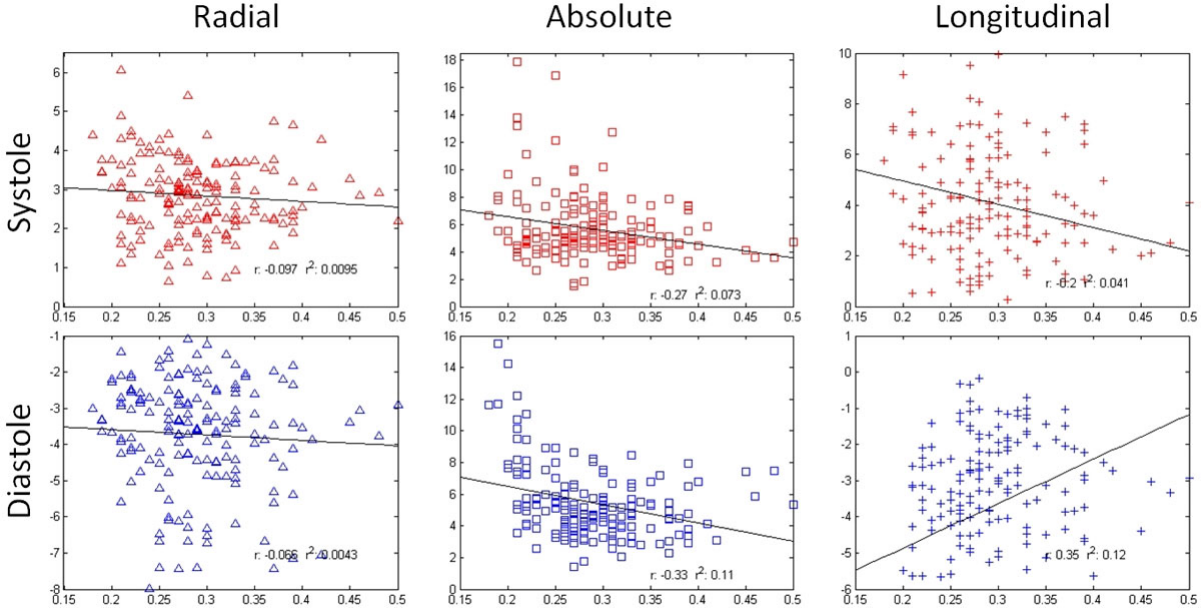
Distribution of myocardial velocities in systole and diastole (radial, absolute, and longitudinal) by ECV fraction. As ECV fraction increases there are reduced longitudinal and absolute myocardial velocites, while little change is observed in radial myocardial velocities.

Eleven patients with suspected non-ischemic cardiomyopathy underwent cardiac MRI (CMR) on a 1.5T magnet (Magnetom Avanto or Aera, Siemens Healthcare, Erlangen, Germany). In addition to the conventional CMR viability protocol, patients underwent both tissue phase mapping (TPM) and T1 mapping with a modified look-locker inversion recovery (MOLLI) technique pre and between 10 and 25 minutes post 0.2 mmol/kg adminstration of an extracellular gadolinium agent. These sequences were performed through the left ventricle in the short axis orientation at basal, mid-chamber, and apical levels. The hematocrit was collected within 48 hours of the CMR to calculate segmental ECV values as described by Kellman, et al., as a marker of microscopic fibrosis. TPM analysis was used to determine the degree of segmental wall motion abnormality. Radial, longitudinal and absolute velocities were measured from the TPM images. Short axis slices were analysed on a segmental basis using the AHA 16 segment model. Segments were classified into four ECV subgroups as detailed in Figure 1; TPM results were compared by ECV subgroup.

## Results

**Figure 2:**
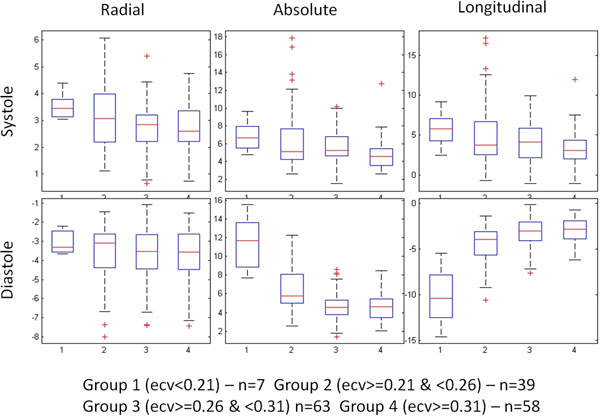
Myocardial segments were grouped into normal (group 1), borderline normal (group 2), borderline abnormal (group 3), and abnormal (group 4) by ECV values as noted above.

Longitudinal and absolute peak systolic and diastolic velocities were reduced in segments with increased fibrosis (Figure 2). These trends are also well-illustrated by ECV subgroup analysis (Figure 1). Most pronounced changes were found for diastolic function with substantial reductions of longitudinal peak velocities (r=0.35). There was little change between radial peak systolic and diastolic velocity with increasing fibrosis in this cohort.

## Conclusions

Our preliminary findings suggest a myocardial structure-function relationship linking myocardial fibrosis with altered myocardial function at a segmental level. Recruitment is ongoing to validate our results in a larger patient cohort.

Kellman et al.: J Cardiovasc MR 2012, 14:63,64

## Funding

Not funded.

